# Selenium Supplementation, Body Mass Composition, and Leptin Levels in Patients with Obesity on a Balanced Mildly Hypocaloric Diet: A Pilot Study

**DOI:** 10.1155/2020/4802739

**Published:** 2020-05-28

**Authors:** Elisabetta Cavedon, Jacopo Manso, Isabella Negro, Simona Censi, Roberto Serra, Luca Busetto, Roberto Vettor, Mario Plebani, Raffaele Pezzani, Davide Nacamulli, Caterina Mian

**Affiliations:** ^1^Familial Cancer Clinic and Oncoendocrinology, Veneto Institute of Oncology IOV-IRCCS, Padua, Italy; ^2^Endocrinology Unit, Department of Medicine, University of Padua, Padua, Italy; ^3^Internal Medicine, Alto Vicentino Hospital, Santorso (VI), Italy; ^4^Internal Medicine 3, Department of Medicine, University of Padua, Padua, Italy; ^5^Department of Laboratory Medicine, University of Padua, Padua, Italy; ^6^AIROB, Associazione Italiana per La Ricerca Oncologica di Base, Padua, Italy

## Abstract

**Background:**

Adequate levels of selenium (Se) have protective effects against several chronic diseases, such as obesity. The aim of this study was to assess the effect of Se supplementation in a selected group of patients with obesity.

**Methods:**

This randomized prospective study included 37 overweight/obese individuals aged 18–65 years, who adopted a slightly hypocaloric diet for 3 months. An intervention group received 240 *μ*g/day of L-selenomethionine for 3 months; a control group received a placebo. Clinical and biochemical parameters, body composition measurements, and the Psychological General Well-Being Index (PGWBI) questionnaire were tested at the beginning and end of the treatment.

**Results:**

A comparison of the two groups showed a significant change in body composition, involving a decrease in body fat mass, between the baseline and the end of the follow-up, in the intervention group. Unlike the placebo group, the group given Se had a significant increase in lean body and muscle mass and a significant decrease in leptin levels after 3 months on diet. At the end of the follow-up, the group given Se scored higher on the PGWBI than those who did not.

**Conclusion:**

Se could reinforce the effects of diet for overweight and obesity. This work was registered in the ISRCTN registry with study ID ISRCTN6106073.

## 1. Introduction

Obesity is rapidly becoming one of the most worrying population health issues. It not only worsens an individual's quality of life, but also is associated with severe medical complications such as diabetes type 2, dyslipidemia, hypertension and other cardiovascular diseases, sleep apnea, various forms of cancer, pulmonary diseases, osteoarthritis, and a consequent increase in mortality/morbidity rates [1].

Leptin is an adiposity signal secreted by the adipocytes proportionally to the amount of body fat. In healthy individuals, it regulates the energy balance by increasing energy expenditure and reducing energy intake [2]. In diet-induced obesity, leptin levels rise due to leptin resistance, a condition deriving from activation of an inflammatory pathway, and from systemic oxidative stress, and involved in skeletal muscle atrophy, a disorder also linked to obesity and inflammation [3, 4]. In fact, obesity is associated with a state of chronic low-grade inflammation, and some studies have found blood concentrations of selenium (Se) inversely correlated with obesity, making Se deficiency a possible marker of adiposity [5–7]. Moreover, depression is also described as an inflammatory condition, like obesity, with which it is often associated [8]. Lower Se blood concentrations correlate with a higher risk of depression and an Se-enriched diet seems to improve the psychoemotional state and mood of obese people [9, 10].

Se is a trace element involved in the proper functioning of the endocrine (and particularly, thyroid) and immune system and capable of modulating the body's inflammatory response due to the antioxidant action of selenoproteins [7]. Selenoproteins include glutathione peroxidases, thioredoxin reductases, and iodothyronine deiodinases. Genetic knock-out studies in mice have demonstrated that at least three selenoproteins are essential as deletion of thioredoxin reductase 1, thioredoxin reductase 2, or glutathione peroxidase 4 results in embryonic lethality [11–14]. In 1996, the World Health Organization (WHO) stated that daily intake up to 400 *μ*g selenium could be considered safe [15]. However, a review by the United States Environmental Protection Agency stated that no adverse effect was seen in adults taking 853 *μ*g/day of Se. Recommended dose of selenium varies in different countries, due to differences in geographical and racial characteristics, besides food habits of that population [16]. Moreover, optimal dose of Se intake in patients with metabolic diseases is difficult to estimate due to the limited amount of studies; however, most of these surveys stated that patients with metabolic disorders might need higher dose of Se than the healthy population (82.4–200 *μ*g) [17].

Taken together, these findings suggest a promising role for Se supplementation in obesity, hence, this pilot study, the aim of which was to assess the effect on body weight and mood of high Se supplementation in a selected group of patients with obesity following a balanced, slightly hypocaloric diet as part of a single-center randomized controlled trial.

## 2. Materials and Methods

### 2.1. Patients and Study Design

**Figure 1:**
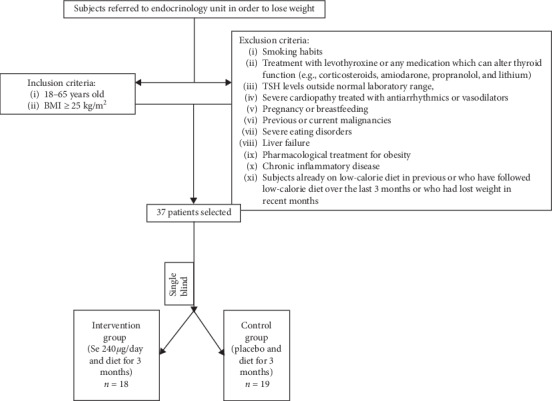
Study design with inclusion and exclusion criteria. Withdrawal due to discovery of pregnancy and changed opinion about the study.

This longitudinal, single-blind, randomized placebo-controlled clinical study involved 37 overweight and obese participants aged 18–65 years referred to our Endocrinology Unit for the purpose of losing weight (Figure 1). All participants had a body mass index (BMI) ≥25 kg/m^2^. The following were reasons for exclusion: smoking habits, treatment with levothyroxine or any medication modifying thyroid function (e.g., corticosteroids, amiodarone, propranolol, and lithium), TSH levels outside the normal laboratory range, severe cardiopathy treated with antiarrhythmics or vasodilators, pregnancy or breastfeeding, previous or current malignancies, severe eating disorders, liver failure, pharmacological treatment for obesity, chronic inflammatory disease, individuals already on a low-calorie diet, or those who had followed a low-calorie diet in the previous 3 months or who had lost weight in recent months.

Participants were divided into two single-blind randomized groups: an intervention group (*n* = 18) took 240 *μ*g/day of L-selenomethionine (S) in a soft gel formula divided into several doses for 3 months and a control group (*n* = 19) received placebo likewise delivered in a soft gel formulation (Figure 1). However, from the intervention group, 2 patients dropped out during the treatment. The reasons were a changed opinion regarding the study and the discovery of pregnancy, respectively.

After an interview with a clinical nutritionist, all participants adopted a balanced, slightly hypocaloric diet for 3 months. They were asked not to change their usual physical activity during the study period. Their clinical parameters and biochemical test findings were examined at the beginning and end of the treatment. Their body composition was measured and their psychological well-being was assessed, using the validated Psychological General Well-Being Index (PGWBI) questionnaire, at the beginning and end of the treatment.

The study was approved by the Research Ethics Committee of Padua University (protocol no. 3220/AO/14). All participants gave their written informed consent before enrolling for the study. This work was registered in the clinical trial ISRCTN registry with study ID ISRCTN6106073.

### 2.2. Clinical and Anthropometric Parameters

The clinical assessment included a general medical examination, recording arterial blood pressure, weight, and height. A participant's height was measured without shoes, with an approximation of 0.5 cm, and body weight was recorded without clothes, with an approximation of 0.1 kg. The BMI was calculated using the formula, weight [kg]/height^2^ [m^2^], and obesity was classified according to the World Health Organization [18].

### 2.3. Biochemical Tests

Serum leptin (expressed as *μ*g/l) was measured at the beginning and at the end of the study using RIA (Radio Immuno Assay).

### 2.4. Body Composition

Body composition was assessed by bioelectrical impedance analysis (BIA) using the BIAVECTOR® (BODYGRAM-AKERN s.r.l. Bioresearch), recording values for lean mass, fat mass, and muscle mass at the beginning and end of the study for each participant.

### 2.5. Types of Diet

A tailored, slightly hypocaloric diet was given to all participants. The quantity of calories was calculated taking into account their basal metabolic rate obtained with the Harris-Benedict equation and reducing each participant's calorie requirement by 20–25%. As recommended by the European guidelines for obesity [19], the composition of the diet was balanced in the contribution of the main macronutrients according to the following criteria:

- Protein intake: 0.8–1.2 g/kg body weight reference/day
- Carbohydrates: 55–65% of total kcal
- Fat to supply the required amount of energy (30%): 10% monounsaturated, 10% polyunsaturated, and 10% saturated

### 2.6. Psychological General Well-Being Index (PGWBI)

The PGWBI is based on a self-report questionnaire comprising 22 items and has proved to be capable of assessing respondents' stress levels [20, 21]. It has been validated and used in many countries in studies on large populations and specific groups. In 2000, the PGWBI was validated in a sample of 1,129 Italian citizens aged 15 years or more, and its normative values are available [20]. The questions cover six areas: anxiety, depressed mood, positive well-being, self-control, general health, and vitality. There are multiple-choice answers with scores ranging from 0 to 5. A total PGWBI score is obtained from the sum of all the items and ranges from 0 to 110. Higher scores indicate greater psychological well-being.

### 2.7. Statistical Analysis

For each continuous variable, a two-tailed Kolmogorov-Smirnov test was first performed to check the type of variable distribution. Then the t-test on independent samples, repeated-measures ANOVA, and the Mann–Whitney and Wilcoxon tests were used, as appropriate, to identify statistically significant differences. All statistical analyses were conducted using the *MedCalc* software bvba, Osted, Belgium (rel. 11.6.0). Values of *p* < 0.05 were considered statistically significant.

## 3. Results

No significant differences emerged between the two groups in terms of age, anthropometric measurements, or body composition at the beginning of the study.  Table 1 shows participants' anthropometric characteristics at the baseline and at the end of the follow-up.

Statistical analysis showed that after 12 weeks, 14/35 (40%) participants had achieved a weight loss of at least 5% from their baseline body weight. Though the difference between the two groups was not statistically significant, the weight loss was more evident in the intervention group, in which 7/16 participants (44%) lost a considerable amount of weight, whereas this was only true of 7/19 (37%) in the placebo group.

There was a statistically significant difference, however, between the changes in body composition in the two groups. Only the intervention group showed a significant reduction in body fat mass from the baseline to the end of the follow-up (*p*=0.0002). The results of ANOVA for repeated measures also showed that the placebo group's proportion of lean body mass did not change after 3 months on a hypocaloric diet, despite their significant body weight loss, whereas the proportion of lean body mass did change in the intervention group (*p*=0.01). However, the difference in this parameter between the two groups did not reach statistical significance at the follow-up.

Considering only muscle mass changes, there were no differences in the placebo group after the hypocaloric diet, irrespective of their body weight, whereas participants in the intervention group showed a significant increase in muscle mass at the follow-up (*p*=0.02).

**Figure 2:**
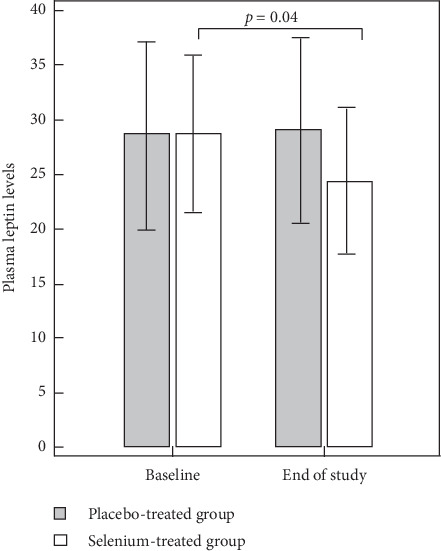
Comparison of plasma leptin levels (*µ*g/l) at the baseline and after the isocaloric diet in the placebo-treated and selenium-treated groups.

Serum leptin levels dropped significantly in the intervention group after the diet (*p*=0.04), while they remained the same in the placebo group (Figure 2).

Interestingly, participants who took Se scored higher on the PGWBI at the follow-up (*p*=0.003), whereas there was no change in the placebo group's scores from the baseline to the follow-up. In addition, among all the participants who obtained a weight loss >5%, the change in their PGWBI scores was much more significant for those who had taken Se (*p*=0.004).

## 4. Discussion

A balanced antioxidant status has a fundamental role in body homeostasis and has been linked to better health outcomes, particularly regarding some manifestations associated with obesity [4]. Selenium is an antioxidant micronutrient available in certain foodstuffs, such as fish, cereals, meat, and vegetables [22–24]. Adequate levels of Se have revealed a protective effect against various chronic diseases, like cancer prevention and cardiovascular protection [25]. Se supplementation exerts its beneficial action by increasing glutathione peroxidase activity and by interacting with inflammatory biomarkers [23, 24].

However, an unexpected result of the Nutritional Prevention of Cancer Trial in the USA was that participants treated with Se (200 *µ*g/day) for an average follow-up of 7.7 years had an increased likelihood of developing type 2 diabetes comparing with a similar cohort of participants taking placebo [26]. However, some issues have to be taken into account: participants were elderly individuals (mean age 63.2 years old) with a self-reported diagnosis of type 2 diabetes and the increased risks for type 2 diabetes among Se-treated participants were no more evident when compared with patients within the top tertile of BMI (>26.76 kg/m^2^), who usually have a dysregulated food habits, with inadequate consumptions of macro- and micronutrients.

Indeed, several studies have reported a positive correlation between serum Se concentrations and dietary Se intake and a negative correlation has been demonstrated between serum Se and BMI [26–28]. This relationship emerged, for instance, in a cross-sectional study conducted on 8- to 13-year-old schoolchildren; those with a BMI above the 85^th^ percentile had a significantly lower dietary Se intake than normal-weight children, after adjusting for energy intake [28]. Data from the 1999 to 2004 US National Health and Nutrition Examination Survey (NHANES) also indicated that children at high risk of overweight were also at greater risk of dietary Se deficiency [29]. Finally, the CODING study, conducted on a large general adult population, clearly indicated that obesity and its severity were associated with a low dietary Se intake: every 1 *µ*g/kg/day increase in dietary Se intake corresponded to a 3–6% decrease in the proportion of body fat mass [30].

The effect of dietary Se intake on body fat composition has been suggested by data emerging from animal interventional experiments. Wang et al. showed that body weight significantly decreased and the ratio of adipose tissue to body weight fell when rats were supplemented with high doses of Se (200 *µ*g/kg/day) [31]. This was due to a lipolytic effect in adipose tissue in parallel with a hepatic storage of free fatty acids. However, two small interventional studies in healthy humans generated contradictory findings [23, 32]. In a population of 54 normal-weight healthy volunteers, Hawkes and Keim found no effect of high-dose Se supplementation (297 *µ*g/day) on body weight composition; in particular, their body fat status changed in the same way as in individuals treated with a low-Se diet (14 *µ*g/day) [32]. More recently, a study conducted by Navas-Carretero et al. showed that the consumption of Se-enriched chickens by 11 individuals did not determine any significant weight loss vis-à-vis a group of 13 individuals who ate chickens not enriched with Se [23]. The authors concluded that this lack of effect of the Se-enriched diet was due to their population having sufficient dietary levels of Se at the baseline and to the low daily dose of Se (36.4 *µ*g/day) added during the trial. In fact, there was no difference in participants' plasma Se levels before and after the supplementation period. The role of Se supplementation in lowering oxidative stress is actually only manifest in populations with endemic Se deficiency, as recently emphasized by several reports [33, 34].

To our knowledge, this is the first placebo-controlled trial of Se supplementation conducted in individuals with obesity. Our findings demonstrate that a high daily dose of Se was able to enhance the effect of a balanced, slightly hypocaloric diet by modifying body composition (reducing fat mass and increasing lean mass) in overweight/obese individuals from an area known to have a moderate Se deficiency, consistently with other reports [15, 22, 24, 35].

For now, the mechanisms behind the beneficial effect of dietary Se on body fat remain largely unclear. There are nonetheless some clues pointing to a link between Se and adipogenesis. Some previous studies applied Se in the differentiation of primary pig and rat preadipocytes and chicken embryonic fibroblasts, suggesting that Se may have a proadipogenic potential [30, 36, 37]. A recent study by Kim et al. showed that Se also inhibits adipogenesis by reducing mRNA expression of peroxisome proliferator-activated receptor-*γ* and fatty acid synthase. On the other hand, Se is capable of activating the expression of transforming growth factor-*β* [38]. But intraperitoneal injections of sodium selenite were able to reduce abdominal fat accumulation and adipocyte size in OLETF rats, stressing the antiadipogenic role of Se in vivo [30,39]. Moreover, Pitts et al. demonstrated that SelM, an endoplasmic reticulum-resident selenoprotein with antioxidant properties, was highly expressed in a hypothalamic area involved in energy metabolism and its deletion resulted in elevated serum leptin levels, increased adiposity, and hypothalamic leptin resistance [11]. On the contrary, in humans, rare mutations in the selenocysteine insertion sequence-binding protein 2, a protein required for selenocysteine incorporation into selenoproteins, were found to determine a multisystem selenoprotein deficiency disorder with paradoxical symptoms with enhanced insulin sensitivity and increased adiposity [40]. In summary, the evidence suggests that certain selenoproteins may act to promote adiposity and insulin resistance, while others may protect against it [11].

In our study, Se supplementation seemed to have a favorable impact on body mass remodeling, suggested by the association between dietary Se intake and a decrease in leptin levels in our Se-treated patients. It is well known that physiological leptin signaling is essential in maintaining body weight. Leptin resistance is a common characteristic of diet-induced obesity, in which anorectic responses to leptin are lower, and hyperleptinemia is a typical finding [4]. The mechanism that leads to leptin resistance is still unclear. Multiple factors, including inflammatory processes and oxidative stress and type of diet, may play a part. Se supplementation could therefore exert beneficial effects not only in reducing peripheral and central leptin resistance (through its antioxidant activity, by increasing selenoproteins activity, and by interacting with inflammatory biomarkers), but also may be via a direct effect on adipose tissue. However, in our survey, no assessments were performed to demonstrate improvement in antioxidant capacity in participants taking Se; for that reason we could only speculate that Selenium's biological activity as an antioxidant could underpin the effects mentioned above.

The glutathione antioxidant system might be implicated in the pathophysiology of mood disorders, too. Support for a role of the glutathione system in psychopathology comes from clinical trials involving treatment with N-acetyl cysteine (NAC), an antioxidant drug precursor of cysteine and glutathione: adjunctive NAC appears to be a safe treatment that has efficacy for schizophrenia [41]. In addition, Se might have protective role against neurodegenerative disease, like Parkinson's disease [42]. An optimal range of serum Se has been found to be associated with a lower risk of depressive symptoms too: higher Se levels correlated with lower scores on a geriatric depression scale and a lower risk of *de novo* major depressive disorders [5]. Prenatal Se supplementation seems to protect against postpartum depression as well [43]. In a double-blind US study involving 11 men confined to a metabolic unit for 120 days, low baseline dietary Se was associated with poor mood; nevertheless, intervention with a high Se diet of 356 *μ*g/day for 99 days did not determine an improvement in mood; it is possible that the psychological effects of being confined may have counteracted any beneficial effects of the Se supplementation [44]. In a trial of 50 British participants, Se supplementation with 100 *μ*g/day for five weeks determined improvement in depression, with a greater effect in individuals with poorer dietary Se intakes [45]. On the contrary, a larger randomised-controlled trial of elderly participants in the UK did not show such effect [46]. Nevertheless, recently Pasco et al. supported the hypothesis that lower dietary Se intakes increase the risk of *de novo* major depressive disorder [42].

In our survey, using a recognized quality of life assessment tool (the PGWBI), our study showed that Se supplementation might improve the mood of overweight/obese participants.

Our study suffers from several limitations that need to be mentioned. First, being a preliminary study, our failure to demonstrate any significantly greater weight loss in the Se-treated patients than in the placebo group may be due to the limited number of patients involved. A further, long-term Se trial on a larger sample, with a sample size calculation, will be needed to confirm our promising preliminary data. In particular, it is fundamental to show that plasma leptin levels continue to drop as the body mass composition improves with Se supplementation. In addition, even if several studies reported a moderate-low Se deficiency status in our country, based on blood Se concentration, in our survey neither data about plasmatic Se levels at the baseline nor food habits, for a semiquantitative estimate of Se intake, were available to confirm Se deficiency in our patient cohorts. Lastly, none of our participants demonstrated any side effects following our brief course of Se supplementation, but it will be necessary to demonstrate that long-standing Se use has no adverse effects in patients with obesity.

In conclusion, our study seems to support the conviction that appropriate dietary Se supplementation may be useful in combating obesity. It could prove a simple and cost-effective intervention for individuals with overweight and obesity.

## Figures and Tables

**Table 1:** Anthropometric and biochemical characteristics of participants at the baseline and at the end of the study.

	Baseline		*p*	End of the study		*p*
	Se-treated group (*n* = 18)	Placebo-treated group (*n* = 19)		Se-treated group (*n* = 16)	Placebo-treated group (*n* = 19)	
Age (ys) ± SD	38 ± 10.9	42 ± 14.4	ns			
Gender			ns			
Male	5	6				
Female	13	13				
Weight (kg) ± SD	106.8 ± 31	114 ± 22.8	ns	103.1 ± 30.7	111.1 ± 21.9	ns
BMI (kg/m^2^) ± SD	37.1 ± 8.9	41.8 ± 8.5	ns	36.9 ± 9.2	40.6 ± 7.7	ns
Fat mass (kg) ± SD	44.2 ± 3.8	47.1 ± 3.9	ns	34.5 ± 3.2	45.4 ± 3.8	0.03
Lean mass (kg) ± SD	62.5 ± 3.7	66.7 ± 2.9	ns	65.9 ± 3.4	66.9 ± 3.2	ns
Leptin levels ± SD	28.9 ± 16.3	28.6 ± 17.7	ns	23.9 ± 15.4	29.4 ± 16.9	0.01
PGWBI score ± SD	71 ± 17	79 ± 16.7	ns	82.3 ± 10	75.1 ± 21.2	0.003

## Data Availability

The data used to support the findings of this study are restricted by the Ethical Committee to protect patient privacy. Data are available from professor Caterina Mian for researchers who meet the criteria for access to confidential data.

## References

[1] Halford J. C. (2001). Pharmacology of appetite suppression: implication for the treatment of obesity. *Current Drug Targets*.

[2] Chan J. L., Heist K., Depaoli A. M., Veldhuis J. D., Mantzoros C. S. (2003). The role of falling leptin levels in the neuroendocrine and metabolic adaptation to short-term starvation in healthy men. *Journal of Clinical Investigation*.

[3] Leon-Cabrera S., Solís-Lozano L., Suárez-Álvarez K., González-Chávez A., Béjar L., Robles-Díaz G. (2013). Hyperleptinemia is associated with parameters of low-grade systemic inflammation and metabolic dysfunction in obese human beings. *Frontiers in Integrative Neuroscience*.

[4] Sáinz N., Barrenetxe J., Moreno-Aliaga M. J., Martínez J. A. (2015). Leptin resistance and diet-induced obesity: central and peripheral actions of leptin. *Metabolism*.

[5] Donma M. M., Donma O. (2016). Promising link between selenium and peroxisome proliferator activated receptor gamma in the treatment protocols of obesity as well as depression. *Medical Hypotheses*.

[6] Saedisomeolia A., Allman-Farinelli M., Hosseini B., Saedisomeolia A., Allman-Farinelli M. (2016). Association between antioxidant intake/status and obesity : a systematic review of observational studies. *Biological Trace Element Research*.

[7] Beckett G. J., Arthur J. R. (2005). Selenium and endocrine systems. *Journal of Endocrinology*.

[8] Soczynska J. K., Kennedy S. H., Woldeyohannes H. O., Yim C. Y., Mcintyre R. S. (2011). Mood disorders and obesity : understanding inflammation as a pathophysiological nexus. *NeuroMolecular Medicine*.

[9] Gosney M. A., Hammond M. F., Shenkin A., Allsup S. (2008). Effect of micronutrient supplementation on mood in nursing home residents. *Gerontology*.

[10] Gao S., Jin Y., Unverzagt F. W. (2012). Selenium level and depressive symptoms in a rural elderly Chinese cohort. *BMC Psychiatry*.

[11] Pitts M. W., Reeves M. A., Hashimoto A. C. (2013). Deletion of selenoprotein M leads to obesity without cognitive deficits. *Journal of Biological Chemistry*.

[12] Jakupoglu C., Przemeck G. K. H., Schneider M. (2005). Cytoplasmic thioredoxin reductase is essential for embryogenesis but dispensable for cardiac development. *Molecular and Cellular Biology*.

[13] Conrad M., Jakupoglu C., Moreno S. G. (2004). Essential role for mitochondrial thioredoxin reductase in hematopoiesis, heart development, and heart function. *Molecular and Cellular Biology*.

[14] Yant L. J., Ran Q., Rao L. (2003). The selenoprotein GPX4 is essential for mouse development and protects from radiation and oxidative damage insults. *Free Radical Biology and Medicine*.

[15] Gerald F. C. (2019). Review article Selenium in global food systems. *British Journal of Nutrition*.

[16] Kieliszek M., Błażejak S. (2016). Current knowledge on the importance of selenium in food for living organisms: a review. *Molecules*.

[17] Wang N., Tan H., Li S., Xu Y., Guo W., Feng Y. (2017). Review article supplementation of micronutrient selenium in metabolic diseases: its role as an antioxidant. *Oxidative Medicine and Cellular Longevity*.

[18] WHO (2000). *Obesity: Preventing and Managing the Global Epidemic. Report of a WHO Consultation*.

[19] Yumuk V., Tsigos C., Fried M. (2015). European guidelines for obesity management in adults. *Obesity Facts*.

[20] Grossi E., Compare A. (2014). Psychological general well-being index. *Encyclopedia of Quality of Life and Well-Being Research*.

[21] Carotenuto A., Fasanaro A. M., Molino I., Sibilio F. (2013). The psychological general well-being index (PGWBI) for assessing stress of seafarers on board merchant ships. *International Maritime Health*.

[22] Nacamulli D., Mian C., Petricca D. (2010). Influence of physiological dietary selenium supplementation on the natural course of autoimmune thyroiditis. *Clinical Endocrinology*.

[23] Navas-Carretero S., Cuervo M., Abete I., Zulet M. A., Martínez J. A. (2011). Frequent consumption of selenium-enriched chicken meat by adults causes weight loss and maintains their antioxidant status. *Biological Trace Element Research*.

[24] Rayman M. P. (2000). The importance of selenium to human health. *The Lancet*.

[25] Flores-Mateo G., Navas-Acien A., Pastor-Barriuso R., Guallar E. (2006). NIH public access: selenium and coronary heart disease: a meta-analysis. *American Journal of Clinical Nutrition*.

[26] Stranges S., Sieri S., Vinceti M. (2010). A prospective study of dietary selenium intake and risk of type 2 diabetes. *BMC Public Health*.

[27] Laclaustra M., Stranges S., Navas-Acien A., Ordovas J. M., Guallar E. (2011). Serum selenium and serum lipids in US adults: national health and nutrition examination survey (NHANES) 2003-2004. *Atherosclerosis*.

[28] Ortega M., Rodríguez R., Aparicio I. (2012). Young children with excess of weight show an impaired selenium status. *International Journal for Vitamin and Nutrition Research*.

[29] Results from the 1999–2004 NHANES Dietary Intake in 2009

[30] Wang Y., Gao X., Pedram P. (2016). Significant beneficial association of high dietary selenium intake with reduced body fat in the coding study. *Nutrients*.

[31] Wang X., Zhang W., Chen H. (2014). High selenium impairs hepatic insulin sensitivity through opposite regulation of ROS. *Toxicology Letters*.

[32] Hawkes W. C., Keim N. L. (2003). Human nutrition and metabolism metabolism in men. *Journal of Nutrition*.

[33] Thomson C. D. (2004). Assessment of requirements for selenium and adequacy of selenium status : a review. *European Journal of Clinical Nutrition*.

[34] Thomson C. D. (2018). Review article selenium and iodine intakes and status in New Zealand and Australia. *British Journal of Nutrition*.

[35] Petricca D., Nacamulli D., Mian C. (2012). Effects of selenium supplementation on the natural course of autoimmune thyroiditis: a short review. *Journal of Endocrinological Investigation*.

[36] Lee K., Hausman D. B., Dean R. G. (1999). Expression of CCAAT/enhancer binding protein C/EBP*α*, *β* and *δ* in rat adipose stromal-vascular cells in vitro. *Biochimica et Biophysica Acta (BBA)-Molecular Cell Research*.

[37] Chen X. L., Hausman D. B., Dean R. G. (1998). Hormonal regulation of leptin mrna expression and preadipocyte recruitment and differentiation in porcine primary cultures of s-v cells. *Obesity Research*.

[38] Kim C. Y., Kim G. N., Wiacek J. L., Chen C. Y., Kim K. H. (2012). Selenate inhibits adipogenesis through induction of transforming growth factor-*β*1 (TGF-*β*1) signaling. *Biochemical and Biophysical Research Communications*.

[39] Kim J. E., Choi S. I., Lee H. R. (2012). Selenium significantly inhibits adipocyte hypertrophy and abdominal fat accumulation in OLETF rats via induction of fatty acid *β*-oxidation. *Biological Trace Element Research*.

[40] Schoenmakers E., Agostini M., Mitchell C. (2010). Mutations in the selenocysteine insertion sequence-binding protein 2 gene lead to a multisystem selenoprotein deficiency disorder in humans. *Journal of Clinical Investigation*.

[41] Zheng W., Zhang Q.-E., Cai D.-B. (2018). N-acetylcysteine for major mental disorders: a systematic review and meta-analysis of randomized controlled trials. *Acta Psychiatrica Scandinavica*.

[42] Pasco J. A., Jacka F. N., Williams L. J. (2012). Dietary selenium and major depression: a nested case-control study. *Complementary Therapies in Medicine*.

[43] Leung B. M. Y., Kaplan B. J., Field C. J. (2013). Prenatal micronutrient supplementation and postpartum depressive symptoms in a pregnancy cohort. *BMC Pregnancy Childbirth*.

[44] Hawkes W. C., Hornbostel L. (1996). Effects of dietary selenium on mood in healthy men living in a metabolic research unit. *Biological. Psychiatry*.

[45] Benton D., Cook R. (1991). The impact of selenium supplementation on mood. *Biological Psychiatry*.

[46] Rayman M., Thompson A., Warren-Perry M. (2006). Impact of selenium on mood and quality of life: a randomized, controlled trial. *Biological Psychiatry*.

